# Femoral Embolization after Cardiac Gunshot

**DOI:** 10.1155/2018/7969845

**Published:** 2018-07-18

**Authors:** Leonardo Toscano, Daniel Terra, Siul Salisbury

**Affiliations:** ^1^Department of Thoracic Surgery, Army Forces Central Hospital, Uruguay; ^2^Department of Emergency, Clinical Hospital, University of Republic, Uruguay; ^3^Department of Thoracic Surgery, Clinical Hospital, University of Republic, Uruguay

## Abstract

Bullet embolism is an uncommon complication from heart gunshot injuries because most of the patients die immediately after trauma. The low frequency of this complication combined with the possible absence of symptoms makes the condition a challenge for the surgeon, delaying diagnostics and leading to severe complications or death. In this case, a small calibre bullet entered the left ventricle and then impacted the femoris artery.

## 1. Introduction

Bullet embolism is a rare, but potentially life-threatening, complication [1, 2]. However, the rise in civilian trauma from low-velocity gunshot wounds increases the likelihood of encountering those kinds of lesions. The coexistence of two fundamental elements—low frequency and the absence of early symptoms—frequently leads to delay in diagnosis that could result in the loss of an extremity or even more severe complications [3, 4].

In cardiac gunshot injuries, only 0.3% of cases present embolization of the distal vessel from the heart [5]. Nearly 81% of patients with cardiac gunshot injury lose their life before arriving at the hospital, with exsanguination being the primary cause of death [6].

## 2. Case Report

A 24-year-old male patient was admitted to the emergency room due to injuries to the left hemithorax as well as a transfixing laceration in the left arm caused by a shotgun of initially unknown calibre.

On examination, the patient was found to be alert and fully orientated. He was hemodynamically stable. His physical examination yielded a small entrance wound from the bullet in his midaxillary line on the left hemithorax at the 4th intercostal space. No exit or other gunshot could be found.

**Figure 1:**
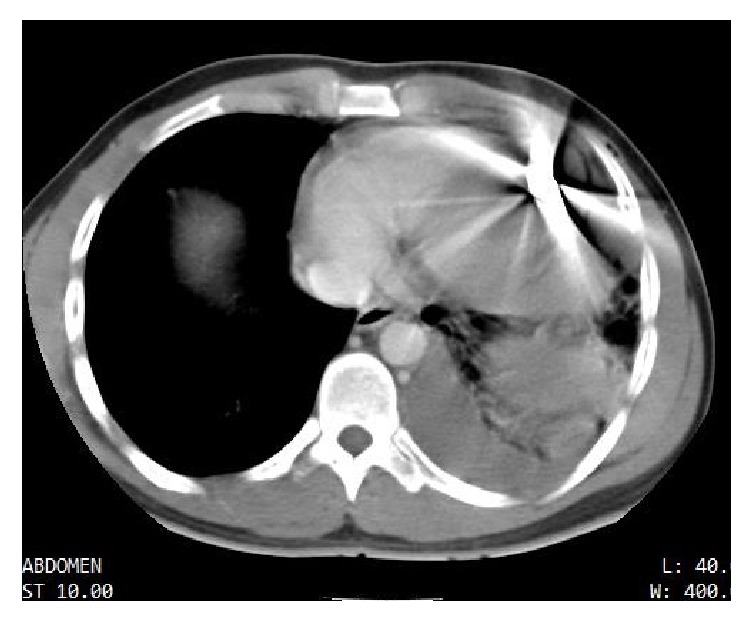
Chest CT at admission shows the bullet near the cardiac apex, not being able to determine if it is inside or outside the pericardial sac.

Computed Tomography (CT) of chest and abdomen showed two rib fractures, a transfixing wound at the lower left lobe, minimal hemothorax, 4 mm pericardial effusion, and foreign metallic body (bullet) in the near left ventricle apex; it was difficult to determine if the metal parts were inside the pericardium or within the musculature of the left ventricle (Figure 1).

Given the risk of cardiac tamponade or cardiac injury, we decided to perform emergency surgery, despite the hemodynamic stability. Surgical access to the thoracic cavity was obtained by left anterolateral thoracotomy; this approach allows handling both pleural cavities in case of other lesions, extending to the other hemithorax.

Following the opening of the cavity, we observed the transfixing left lower lobe lesion with bone fragments, as well as a moderate amount of blood and clots in the pleural cavity (300cc approx.). Also, a hematoma could be spotted in the pericardial fat.

After pericardiotomy, we found a small amount of blood and noticed a small hole in the anterior wall of the left ventricle, without bleeding. Since we could not find the bullet, we decided to perform a radioscopy to determine its location, but we were unable to find it inside the thorax.

The cardiac lesion was repaired by separate sutures “U” with polyester suture line 2-0 and the lung segment resected with a mechanical suture. After repair of the injuries and review of the hemostasis, one drain was inserted.

Once the patient was at the Intensive Care Unit in stable clinical condition, we performed a Transoesophageal Echocardiography that showed a normal cardiac function, but the bullet was not seen.

**Figure 2:**
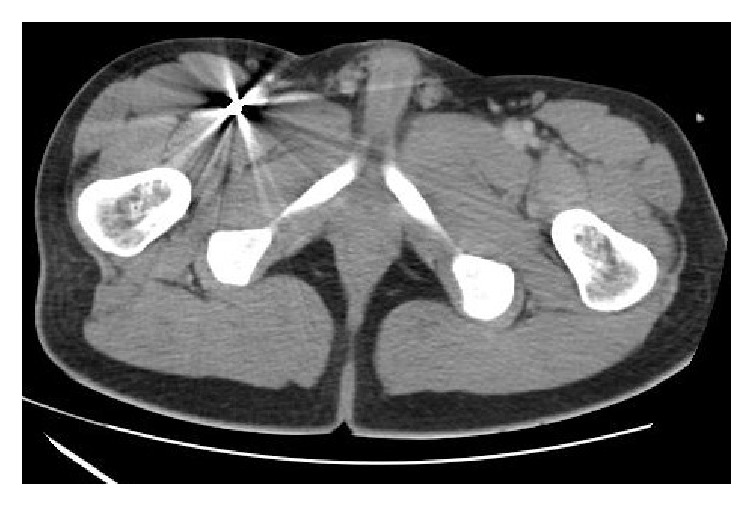
24 hrs after admission, body CT shows the bullet at division of femoris artery.

**Figure 3:**
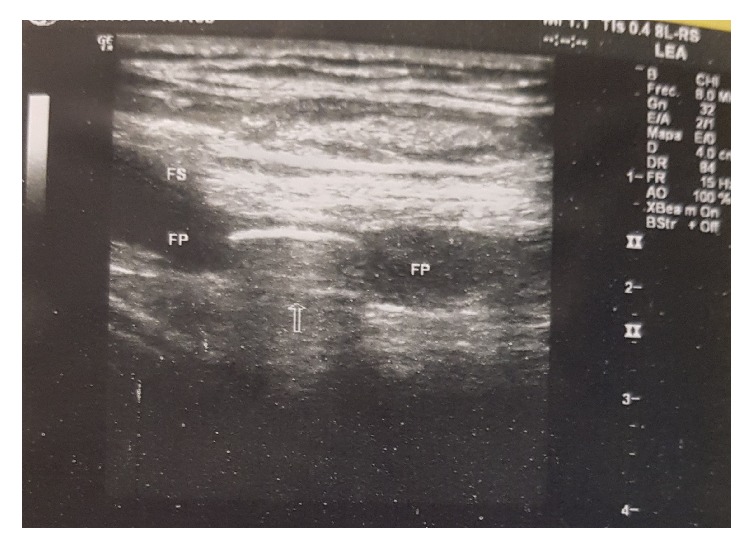
Lower right limb ultrasound shows the bullet at division of femoris artery, with obstruction of profundal femoris artery.

We decided to perform a body CT that identified the bullet at the division of the femoris artery (Figure 2); this was confirmed by a Doppler ultrasound that revealed a lumen obstruction of the profundal femoris artery (Figure 3). The vascular team decided to perform surgery and removed a 0.38 calibre bullet, with no complications. The patient was discharged 15 days after the operation.

## 3. Discussion

“The occurrence of free projectiles in the bloodstream, although doubtless very rare, has already become something more than a surgical curiosity, and its possibility may well be borne in mind by those who observe anomalous symptoms after gunshot wounds, especially when the projectile is not found” [7].

A century after this statement was written, it continues to be of great help for the diagnosis of this low incidence complication. In 1834, Thomas Davis reported the first bullet embolus and was cited by Bland-Sutton in 1919 [8]. One of the biggest papers on heart injuries written by Rich et al. analysed 7,500 vascular trauma cases occurring during the Vietnam war [9]. The findings of only 22 cases (0.3%) were complicated by foreign-body emboli.

Injuries to the heart present with signs of major blood loss or cardiac tamponade, which determines the patient's death at the incident site [6]. There are two ways for foreign objects to penetrate the vascular tree (arterial or venous): by direct entry into the lumen or by the erosion of the vessel wall [10]. This embolization could be arterial or venous (80% versus 20%), and the embolism could occur at the moment of the impact or take place hours or days after the impact [11].

The diagnosis of bullet embolization should be suspected in a wounded patient with no exit wound if, on X-ray, no bullet in the area of injury or signs of limb ischemia are detected [2].

Two conditions must be present at the time of the impact for those kinds of embolization to develop. First, at the precise moment that the bullet passes into the heart, it must have a low kinetic energy in order to penetrate but not trespass the vessel or the heart. Second, the bullet must have a diameter less than the blood vessel to travel through the bloodstream [1]. In the crossing through the soft tissue and the bone tissue, there is a loss of kinetic energy that allows that penetration. Once inside the bloodstream, the blood flow (with major kinetic energy) moves the bullet to a distal site.

The bullets of low calibre—0.38 in our case—do not possess great kinetic energy, which allows them to penetrate the left ventricle, to be dragged into the bloodstream and embolized in the superficial femoral artery.

The embolic site was ultimately determined by missile size. Strauss [12] suggested that contributory factors may include the patient's position following wounding, respiratory activity, hemodynamics, and gravity. There is a high incidence of embolization to the left lower extremity, which was twice as high as that to the right (this was related to the lesser angle at which the left iliac artery originates from the aortic bifurcation) [13].

Without symptoms of limb ischemia, conservative management of impacted bullet has been reported [1]; however, embolectomy is the gold standard in the management of peripheral bullet arterial emboli [1]. The presence of an impacted bullet produces ischemia and infarction in most cases, although other complications such as the formation of a proximal clot, infection, and eventually death can also occur [14].
